# Bees in the six: Determinants of bumblebee habitat quality in urban landscapes

**DOI:** 10.1002/ece3.8667

**Published:** 2022-03-21

**Authors:** Ida M. Conflitti, Mohammad Arshad Imrit, Bandele Morrison, Sapna Sharma, Sheila R. Colla, Amro Zayed

**Affiliations:** ^1^ 7991 Department of Biology York University Toronto Ontario Canada; ^2^ 7991 Faculty of Environmental & Urban Change York University Toronto Ontario Canada

**Keywords:** foraging distance, landscape features, nest density, pollinator conservation

## Abstract

With growing urbanization, it is becoming increasingly important to design cities in a manner that sustains and enhances biodiversity and ecosystem services. Native bees are critical pollinators that have experienced substantive declines over the past several decades. These declines have captured the attention of the public, particularly urbanites, prompting a large interest in protecting pollinators and their habitats in cities across North America and Europe. Unfortunately, we currently lack research about specific features of urban environments that can enhance the fitness of pollinators. We carried out an intensive study of *Bombus impatiens*, the Common Eastern Bumblebee, in the city of Toronto (Canada's largest city), to better understand landscape parameters that provide high‐quality habitat for this species and likely other generalist bees. We divided the city into 270 grid cells and sampled a large number of worker bees, which were then genotyped at twelve hypervariable microsatellite loci. The genetic data allowed us to quantify the effective number of colonies and foraging distance for bumblebees in our study area. We then asked how the city's landscape and human population demography and income are associated with the availability of high‐quality habitat for *B*. *impatiens*. Several aspects of Toronto's landscape influenced colony density and foraging range. Urbanization had a clear effect on both colony density and foraging distance of workers. On the other hand, functional (i.e., not cosmetic) green space was often associated with higher quality habitats for bumblebees. Our study suggests several planning strategies to enhance habitat quality for bumblebees and other pollinators in cities.

## Graphical abstract

Population genetic analysis of several hundred bumblebees in the city of Toronto indicated that urbanization and the availability of functional (i.e., not cosmetic) green space influenced habitat quality for the common Eastern Bumblebee and likely other generalist pollinators. Our study suggests several planning strategies to enhance habitat quality for bumblebees and other pollinators in cities.

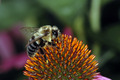

## 1 INTRODUCTION

Approximately 55% of the world's human population live in urban areas, and this is expected to grow to nearly 70% by 2050 (UN, 2018). Urban environments can sustain a large number of native, and sometimes at‐risk, species thereby providing considerable value for conserving biodiversity and ecosystem services (Nilon et al., 2017). Moreover, cities contain a substantive proportion of the world's human population that—if exposed to nature and wildlife—can act as catalysts and ambassadors for species conservation. However, it is not always obvious how to design cities in a manner that best supports biodiversity and ecosystem services (Lepczyk et al., 2017).

There has been a growing concern over the decline of bees across the globe (Cameron et al., 2011; Grixti et al., 2009; Potts et al., 2010; Powney et al., 2019; Senapathi et al., 2015). These declines have captured the attention of the public, leading to widespread interest in conserving and protecting bee populations. The free ecosystem services provided by wild bees in cities are important for continued pollination of community vegetable gardens, native plant gardens, indigenous medicine gardens, naturalized green spaces, urban agricultural fields, and fruit trees. Cities can host diverse pollinator assemblages (Hall et al., 2017), and several major North American and European cities have implemented mandates or legislation to protect pollinators. Indeed, cities can now seek pollinator‐friendly certifications through programs such as “Bee City” (e.g., beecitycanada.org and beecityusa.org) by pledging to implement a suite of actions aimed at protecting pollinators. While some of the general recommendations for protecting pollinators in cities make intuitive sense, they are not often backed by research. Planting “pollinator gardens” is often the top recommendation but we note that its efficacy in improving bee health is rarely empirically demonstrated (but see Baldock et al., 2019; Leve et al., 2019). Installing “bee hotels” (i.e., fixtures with hollow tubes for bees to nest in) was a common recommendation for conserving pollinators in cities but it has since been shown to not benefit native bees (MacIvor & Packer, 2015). We need research to understand which features of urban environments enhance pollinator health to help inform bee conservation initiatives in cities.

Several studies in mixed environments have identified the importance of landscape as a driver for bee diversity and habitat quality. For example, sampling twelve sites in urban, seminatural, and agriculture areas in France, Geslin et al. (2016), discovered that “urbanization”—quantified as proportion of impervious surfaces within a site—was negatively associated with bee abundance and species richness. Similar patterns were observed within farms and gardens in the greater Chicago metropolitan area (Bennett & Lovell, 2019). Using genetics to infer the location and density of bumblebee colonies, Goulson et al. (2010) discovered that the size and number of gardens within agricultural landscapes had positive effects on the survival of two bumblebee species in the UK. Another UK study carried out in agricultural landscapes found that forage patches sown with flower mixtures had a positive influence on bumblebee species richness and worker densities (Carvell et al., 2011). Jha and Kremen (2013a) found that floral diversity across mixed landscapes (i.e., crops, grasslands, orchards, riparian forests) was associated with the foraging distance for the bumblebee *Bombus vosnesenskii*. In the same study, the authors found that the proportion of paved surfaces had a negative influence on *Bombus vosnesenskii's* nest density. The above studies in mixed or agricultural landscapes suggest bee habitat quality is enhanced by the availability of floral resources and is negatively impacted by impervious paved surfaces. However, it still remains to be demonstrated if the same drivers of habitat quality identified in studies of mixed or agriculture landscapes still operate at the *within*‐*city* scale that is needed to inform urban planning.

In addition to physical features, the characteristics of the human populations inhabiting urban landscapes can potentially influence the quality of bee habitats via a phenomenon known as the “luxury effect” (Leong et al., 2018); wealthier neighborhoods may actively or passively provide higher quality habitats for wildlife than poorer neighborhoods. A recent study discovered that higher household income was positively associated with pollinator abundance in the UK (Baldock et al., 2019), although household income was not associated with bee abundance, richness, or community composition in Chicago (Lowenstein et al., 2014). Understanding the importance of human population demography more broadly in pollinator conservation will be critical for ensuring that ecosystem service provisions in cities are equitable.

**FIGURE 1:**
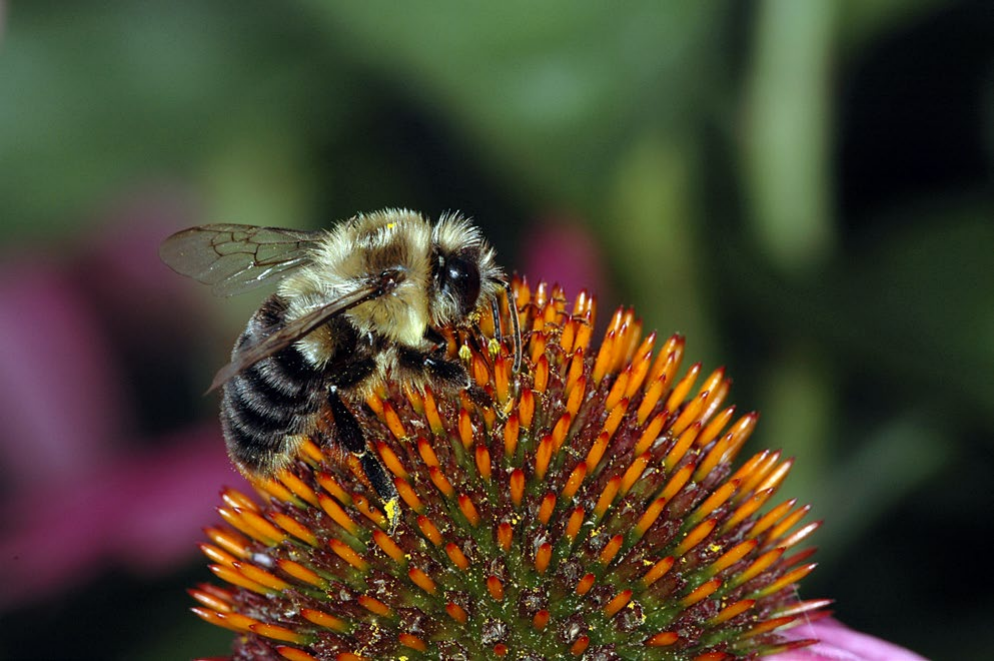
A common eastern bumblebee, *Bombus impatiens*, worker foraging on purple coneflower (*Echinacea* sp.). Photograph by Amro Zayed

Here, we leveraged Toronto's (Ontario, Canada) rich open data resources to study how the physical features of the city and the demography of its human population influence habitat quality for bumblebees. Toronto is the capital city of the province Ontario and, with an estimated population size of over 6 million people in the city and its surrounding suburbs (Statistics Canada, 2020), is the largest city in Canada and among the top ten largest cities in North America. We studied the Common Eastern Bumblebee *Bombus impatiens* (Williams et al., 2014) as it has many traits that may allow us to generalize our findings to other bumblebees and native bees (also see the Discussion section): (1) *B*. *impatiens* (Figure 1) is common throughout the city, thereby allowing us to explore the relation between landscape features and bee habitat quality across a large swathe of Toronto; (2) *B*. *impatiens* is polylectic (i.e., pollen generalists) like the majority of bees found in cities (MacIvor et al., 2014; Matteson et al., 2008); (3) *B*. *impatiens* visits many plant species that are known to be attractive to a wide range of native bee species (Colla & Dumesh, 2010; Gervais et al., 2020; Vaudo et al., 2014); and (4) *B*. *impatiens* is social with adults active from early spring to late fall (Colla & Dumesh, 2010), thereby increasing the temporal scope of our analysis. While *B*. *impatiens* is certainly one of the largest bees found within Toronto, the reasons described above still suggest that it can act as a proxy for a large number of generalist bees inhabiting the city.

To study how Toronto's landscape influences *B*.* impatiens*, we first divided the city into 270 2 × 2 km grid cells and then randomly sampled *B*. *impatiens* workers from across the city. The sampled workers were genotyped allowing us to estimate the number of colonies, and how far workers travel to forage, across the different grid cells. We then carried out analyses to examine which physical and demographic features were associated with habitat quality for *B*. *impatiens*. In lieu of direct data on reproductive fitness, we indirectly gauged high‐quality habitat as features that reduce foraging distances and increase colony density within grid cells. Foraging is metabolically costly which can directly influence the lifespan of workers and fitness of colonies (Kelemne et al., 2019; Rueppell et al., 2007; Tomlinson et al., 2017). Areas with ample floral resources nearby should thus lessen the metabolic costs of foraging allowing bumblebee colonies to allocate more resources toward reproduction, thereby increasing colony fitness. Similarly, we expected areas with more floral resources in urban environments to sustain more bumblebee colonies (i.e., greater colony density per unit area), as previously found in agricultural and mixed‐use landscapes (Osborne et al., 2008).

## 2 METHODS

### 2.1 Population sampling

**FIGURE 2:**
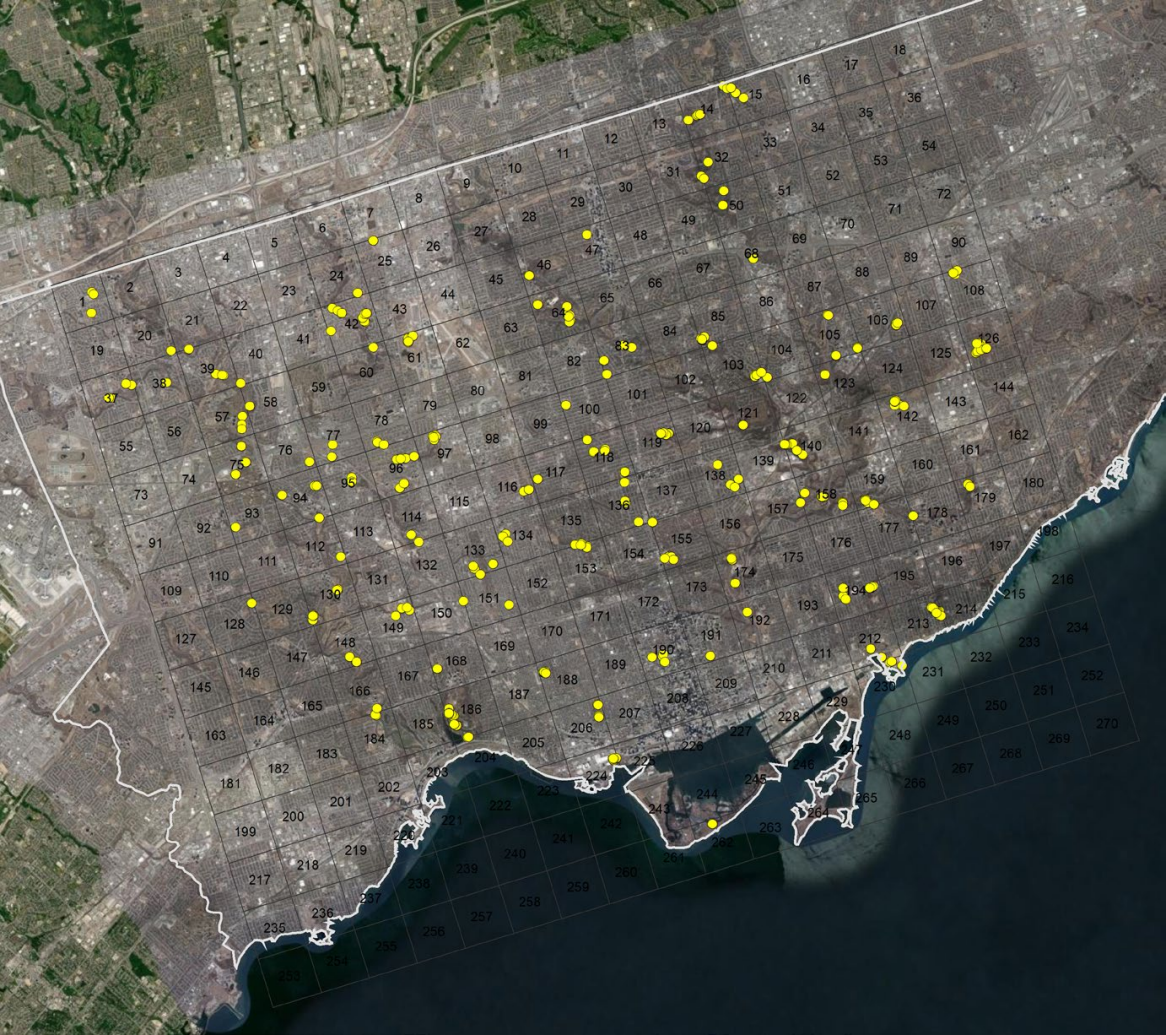
Map of the City of Toronto showing 2 × 2 km grid cells and bumblebee sampling sites. Sampling locations are represented by yellow circles. Please see SI Dataset 1 for exact sampling coordinates

The city of Toronto (Ontario, Canada) was divided into 270 grid cells, each measuring 2 × 2 km (4 km^2^) (Figure 2). Grid cells were selected at random and visited for sampling between July and October 2016—this period is within *B*. *impatiens*’ worker activity period in Southern Ontario (Colla & Dumesh, 2010). In total, we collected 760 specimens from 86 grid cells, across 27 collection days (Data S1). At the start of a collecting day, a random number generator was used to pick a focal grid cell for sampling. We typically traveled within a grid cell and netted flying bumblebees as they foraged on flowers. While we tried to sample bees from many different locations within a grid cell, this was not always possible in highly urbanized areas where green space (and flowers) is sparse. After field identification, *B*. *impatiens* workers were collected and kept in a cooler. After either 2 h or approximately 60 specimens were collected from a single grid cell, the collector would then move to an adjacent grid cell for collecting. This process of visiting adjacent grid cells helped us maximize the number of grid cells sampled in a given day by minimizing travel time. At the end of the sampling day, bees were transported to York University's Keele Campus and stored in a deep freezer at −80°C. The exact sampling location where each individual was caught was recorded for downstream analysis.

### 2.2 DNA extraction

We extracted DNA from bee tissues using the Mag‐Bind^®^ Blood & Tissue DNA HDQ 96 Kit (Omega Bio‐tek Inc.) optimized for the KingFisher™ Flex Purification System (Thermo Fisher Scientific Inc.). Briefly, one half of a bee's thorax was finely ground in a 1.5 ml microcentrifuge tube in liquid nitrogen. Tissue lysis buffer (350 µl; Omega Bio‐tek Inc.) and Proteinase K (20 µl; Omega Bio‐tek Inc.) were added, and the tube was vortexed for 10 s, followed by an overnight incubation at 50°C. Samples were then centrifuged at 7000 *g*/rcf for 10 min. The supernatant (approx. 300 µl) was transferred to a new tube and centrifuged similarly for another 10 min. We then followed the kit's published protocol for extracting DNA using the KingFisher™ Flex Purification System. The eluted purified DNA (100 µl) was used for microsatellite genotyping.

### 2.3 Microsatellite genotyping and scoring

We used fluorescently labeled primers (Table 1) to genotype 12 hypervariable microsatellite loci previously identified for bumblebees (Estoup et al., 1995, 1996; Funk et al., 2006). Each locus was tested for usability in a two‐step process on a small number of *B*. *impatiens* workers. First, a temperature gradient was run, with the polymerase chain reaction (PCR) conditions described below, to determine optimal annealing temperatures and product sizes. The gradients were set to 3–5°C above and below the temperatures listed in Estoup et al., 1995, Estoup et al., 1996, and Funk et al., 2006. PCR products were run on 2% agarose gels and genotyped (see below). Agarose gel results and chromatograms were used to group primers for multiplexing (i.e., multiple loci amplified in a single PCR reaction) or poolplexing (i.e., loci amplified separately and combined for genotyping) based on annealing temperatures, product sizes, and product intensities. Next, the forward primers in each group were labeled with a different color fluorescent tag and each group of multiplexed or poolplexed loci was tested for usability via PCR and genotyping. After optimization and testing, the 12 primers were amplified as 3 multiplex sets (each with 2 primers) and 2 poolplex sets (each with 3 primers) as shown in Table 1.

**TABLE 1:** Primers, annealing temperatures (*T*_m_), population genetics estimates, and groups used for PCR multiplexing and poolplexing.

Locus	Label	Primer Sequence (5’–3’)	*T*_m_ (°C)	Pooling Volume (μl)	Number of alleles	Size range of alleles	Observed heterozygosity	Expected heterozygosity
Multiplex groups								
BT01^b^	F‐FAM	CCGATCTGTGAGAATGACAGTATCG	53.5	N/A	18	143–201	0.800	0.796^a^
	R	CGTGTTTCGATTAGCAAAGCTACG						
BT23^b^	F‐AT5	GCAACAGAAAATCGTCGGTAGTG			14	158–206	0.427	0.428^a^
	R	GCGGCAATAAAGCAATCGG						
BT08^c^	F‐AT0	AGAACCTCCGTATCCCTTCG	52.5	N/A	12	156–182	0.664	0.643^a^
	R	AGCCTACCCAGTGCTGAAAC						
BT26^c^	F‐AT5	AGCGGGACCTGGTAAAAACG			19	93–155	0.886	0.887
	R	CGATTCTCTTCGTGGTCAGTTCTCC						
B124^d^	F‐HEX	GCAACAGGTCGGGTTAGAG	56.5	N/A	30	226–306	0.890	0.915
	R	CAGGATAGGGTAGGTAAGCAG						
B126^d^	F‐FAM	GCTTGCTGGTGAATTGTGC			20	136–182	0.863	0.871
	R	CGATTCTCTCGTGTACTCC						
Poolplex groups								
B96^e^	F‐HEX	GGGAGAAAGACCAAG	49	5.0	19	228–266	0.780	0.775
	R	GATCGTAATGACTCGATATG						
BL13^e^	F‐FAM	CGAATGTTGGGATTTTCGTG	53	2.5	15	144–194	0.601	0.618
	R	GCGAGTACGTGTACGTGTTCTATG						
BL15^e^	F‐AT0	CGAACGAAAACGAAAAAGAGC	52	2.5	20	117–173	0.862	0.853
	R	TCTTCTGCTCCTTTCTCCATTC						
B10^f^	F‐FAM	GTGTAACTTTCTCTCGACAG	52	4.5	20	171–229	0.806	0.806
	R	GGGAGATGGATATAGATGAG						
BT10^f^	F‐HEX	TCTTGCTATCCACCACCCGC	57	3.5	27	135–189	0.911	0.923
	R	GGACAGAAGCATAGACGCACCG						
BTERN01^f^	F‐AT5	CGTGTTTAGGGTACTGGTGGTC	54	2.0	22	98–162	0.802	0.799
	R	GGAGCAAGAGGGCTAGACAAAAG						

^a^Loci that deviate from Hardy–Weinberg equilibrium after Bonferroni correction (*p *< .004). Superscripts b to f denote combinations of primers that were multiplexed or poolplexed together.

All PCRs were performed using a Mastercycler (Eppendorf, Canada) in either strip tubes or 96‐well plates with one negative control, where sterile water was used instead of DNA. Each 25 µl PCR consisted of 0.5 µl of each 10 µM primer (Integrated DNA Technologies Inc.), 12.5 µl Taq 2X Master Mix (New England Biolabs Lt.), 1.5 µl DNA, and nuclease‐free water (Thermo Fisher Scientific Inc.). PCRs were carried out with an initial denaturation step of 94°C for 3 min; followed by 35 cycles of 94°C for 30 s, *T*_m_ (Table 1) for 30 s and 72°C for 30 s, with a final incubation at 72°C for 10 min. Following multiplexing and poolplexing, 10 µl samples were sent to The Center for Applied Genomics for genotyping using an ABI3730xL DNA Analyzer (Applied Biosystems) with GeneScan 500 LIZ Size Standard (Applied Biosystems). Geneious v11.1.2 (Biomatters Limited) was used to view chromatograms and to automatically bin alleles and assign genotypes. All assigned bins and genotypes were manually checked for accuracy and adjusted where needed. Micro‐checker v2.2.3 (van Oosterhout et al., 2004) was used to identify potential sources of genotyping error. All loci passed Micro‐checker's quality control steps with no evidence of scoring error due to stuttering, large allele drop‐out, or null alleles.

### 2.4 Population genetic analyses

We used Colony v2.0.6.4 (Jones & Wang, 2010) to partition worker bees into full sister groups using the following parameters: (1) haplodiploidy; (2) monogyny and single queen mating; and (3) 0% allele dropout rate and 1% rate for other genotyping errors, including mutations (based on our microchecker results). We ran this analysis 3 times using long runs to ensure convergence of the sibship reconstructions. Following established methods (Carvell et al., 2017; Dreier et al., 2014), we retained sibship clusters with probabilities greater than or equal to 80%; this resulted in 303 worker bees grouped into 118 clusters, each containing 2 or more sisters, and 457 singletons (i.e., worker bees that did not have any sisters in our dataset).

We used this dataset to estimate the effective number of colonies and average foraging distance for each grid cell following established methods (Carvell et al., 2017; Dreier et al., 2014; Redhead et al., 2016), as described below. Here, we decided to exclude all 457 singletons from further analysis because we cannot be certain if they resided in a grid cell, or were simply foraging in a grid cell. Moreover, of the 118 sibship groups detected, 31 (totaling 79 worker bees) contained only sister bees with the same geographic coordinates. These groups were also removed from further analysis because they could not be used to calculate foraging distance using our method of triangulation (see below). The remaining 87 sibship groups (totaling 224 worker bees), containing sister bees having 2 or more different (i.e., not identical) coordinates, were mapped in ArcGIS v10.6 (Esri, USA).

Following established methods (Carvell et al., 2017; Dreier et al., 2014; Redhead et al., 2016), colony locations were calculated as the mean center of the sampling coordinates for all sister bees belonging to a sibship group, and the total number of colonies were tallied for each grid cell. However, the total number of colonies we detected for each grid cell represents a minimum. Therefore, we followed Charman et al. (2010) to calculate the effective number of colonies (colNe, i.e., minimum number of sampled + unsampled colonies per grid cell) for cases where low numbers preclude reliable estimates using a Poisson distribution: colNe in social haplodiploids = (4.5 Nmn)/(1 + 2 m), where *N* = number of colonies, m = mating frequency, and *n* = number of queens per colony (Charman et al., 2010). Following Charman et al. (2010), colNe in each grid cell was calculated as 1.5 × the total number of colonies in that grid cell, assuming monogyny and monoandry. The first assumption is clearly supported as polygyny is extremely rare in bumblebees (Cameron & Jost, 1998), and the available genetic data on *B*. *impatiens* have not detected nests with multiple queens (Payne et al., 2003). While multiple mating is known to occur in *B*. *impatiens*, it is relatively rare and the average effective number of mates per queen (1.06) is close to the value expected with monogamy (1) (Payne et al., 2003). Rare cases of multiple mating would lead to undetected half‐sib relationships, but we do not expect this to lead to confounds in the downstream analysis. To calculate average foraging distance (aveMeanFD), colony‐specific worker foraging distances were calculated as the mean of all the Geodesic (straight‐line) distances to a colony for all sister bees belonging to that colony (even if the sisters were captured in different grid cells). Then, grid cell values were calculated as the average of all the foraging distances for colonies located in a grid cell.

We used PopGenReports (Adamack & Gruber, 2014) to estimate basic population genetic parameters (e.g., number of allele, observed and expected heterozygosity) and to test for deviations from Hardy–Weinberg equilibrium at each locus. For this analysis, our dataset was pruned by randomly retaining one sister from each colony that contained 2 or more sister bees. We used a Bonferroni correction when assessing significant departures from Hardy–Weinberg equilibrium.

### 2.5 GIS and City of Toronto landscape layers

Twenty‐eight physical and demographic variables were extracted from 10 maps obtained from the following sources: (1) ArcGIS Online, https://www.arcgis.com/home/index.html; (2) City of Toronto Open Data Portal, https://open.toronto.ca/; (3) Ontario GeoHub, https://geohub.lio.gov.on.ca/; and (4) Toronto and Region Conservation Authority Open Data Portal, https://data.trca.ca/ (Table 2). All maps were transformed to World Geodetic System 1984 geographic coordinate system in ArcGIS. Data were extracted for each grid cell as follows: (1) count data: calculated as the number of occurrences of each variable in a grid cell; (2) percent data: calculated as the percent of the total area of a grid cell covered by each variable; (3) weighted average data: for some maps, single value data were available for various sized and shaped polygons covering Toronto (e.g., population parameters were given for 140 polygons representing Toronto neighborhoods). In these cases, data were calculated as the average value of all the polygons covering a grid cell weighted by the percent area of the grid cell covered by each of the polygons (Table 2).

**TABLE 2:** Physical and demographic variables extracted from GIS maps

Variable	Description	Source^1^, ^2^
Physical features		
buildPerc	Percent of grid cell covered by buildings	1a
roofPerc	Percent of grid cell covered by green roofs	1b
green1Perc	Percent of grid cell covered by city parks	1c
treeCount	Number of city‐owned trees located on road allowances per grid cell	2d
treeCanopyPerc	Percent of grid cell covered by tree canopy	2e
grassShrubPerc	Percent of grid cell covered by grass or shrub. In Toronto, this mostly represents grass lawns.	2e
waterPerc	Percent of grid cell covered by water	2e
bareEarthPerc	Percent of grid cell covered by bare earth	2e
roadPerc	Percent of grid cell covered by roads	2e
otherPavedPerc	Percent of grid cell covered by paved surfaces (excluding roads and buildings)	2e
elevat	Weighted average elevation per grid cell	3f
slope	Weighted average slope per grid cell	1g
beachPerc	Percent of grid cell containing beach‐bluff in watersheds	4h
forestPerc	Percent of grid cell containing forest in watersheds	4h
meadPerc	Percent of grid cell containing meadows in watersheds	4h
succPerc	Percent of grid cell containing successional habitat in watersheds	4h
wetPerc	Percent of grid cell containing wetlands in watersheds	4h
Demographic features		
popTotalCom	Weighted average total population per grid cell	1i
popMale	Weighted average number of males per grid cell	1i
popFemale	Weighted average number of females per grid cell	1i
pop_less20	Weighted average population less than 20 years old per grid cell	1i
pop_20‐39	Weighted average population from 20 to 39 years old per grid cell	1i
pop_40‐59	Weighted average population from 40 to 59 years old per grid cell	1i
pop_60plus	Weighted average population 60 years of age or older per grid cell	1i
popDensity	Weighted average population density per grid cell	1g
houseDensity	Weighted average household density per grid cell	1g
indTI	Weighted average total income for individuals per grid cell	1j
famTI	Weighted average total income for households per grid cell	1j

^1^ Web source: (1) ArcGIS Online, https://www.arcgis.com/home/index.html; (2) City of Toronto Open Data Portal, https://open.toronto.ca/; (3) Ontario GeoHub, https://geohub.lio.gov.on.ca/; and (4) Toronto and Region Conservation Authority Open Data Portal,https://data.trca.ca/.

^2^ Map name: (a) Toronto building polygons; (b) Toronto green roof permits; (c) Toronto greenspace; (d) Street tree; (e) Forest and land cover; (f) Greater Toronto area digital elevation model; (g) Toronto slope; (h) Habitat; (i) Toronto neighborhoods; and (j) Toronto profile of income by dissemination area (i.e., small geographic units with approximately 400 to 700 persons).

### 2.6 Statistical analyses

We were able to estimate the effective number of colonies and average foraging distances within 49 2 × 2 km grid cells in Toronto (Data S1). We first examined the distribution of the population genetic and landscape parameters and found that transforming the average foraging distance using natural log improved normality (Shapiro–Wilk normality test, *p* = .07). To understand the relationships between Toronto's landscape and demographic variables (Table 2) and effective number of *B*. *impatiens* colonies and average log foraging distance, we performed Spearman correlations using the library Hmisc (Harell, 2021) and corrplot (Wei & Simko, 2017) in R. We corrected *p*‐values for multiple testing using the Benjamini–Hochberg false discovery rate (FDR) adjustment (Benjamini & Hochberg, 1995). We report adjusted p‐values in the results section.

We also performed multiple linear regressions using statsmodels (Seabold & Perktold, 2010) to understand how landscape and demographic features of Toronto influence bumblebee effective nest density and average log foraging distances. We first selected the models with the highest adjusted *R* ^2^ and then selected the most parsimonious model using the Akaike information criterion, AIC (Burnham & Anderson, 2002), where the lowest value is the most parsimonious model. For the most parsimonious models, we then used the function varpart from the vegan package (Oksanen et al., 2019) to partition the variation between the different explanatory variables.

We then performed a redundancy analysis (RDA) to explore the association between log‐transformed foraging distance and number of unique bumblebee colonies with landscape and human demographic variables, using the “vegan” package in R (Oksanen et al., 2019). RDA is a direct gradient ordination approach that allows us to summarize the linear relationship between our explanatory variables and a multivariate set of response variables (Rao, 1964). To account for multicollinearity between explanatory variables, we calculated correlations between variables and used the variance inflation factor (VIF>5) (Dormann et al., 2013) to remove variables that were highly correlated to others. A number of multicollinear variables (tree canopy cover, % city parks, % forests in watersheds, % other paved surfaces, total human population, human population over 60 years old and human population between 40 and 59 years old) were retained in our final model as they were biologically relevant to our study and cumulatively explained significant amounts of variation. We decided to retain these multicollinear variables because removing them would make the biological interpretation and actionable implications of our analysis more difficult. We used a permutation test to determine global significance of the RDA model (Borcard et al., 2011).

Moreover, we used the function “varpart” in the “vegan” package (Oksanen et al., 2019) to partition the variation explained by three broad categories of explanatory variables: (1) human population and demographic factors, (2) human‐made infrastructure, and (3) natural habitat. After removing multicollinear variables, the human population and demographic category comprised population density, total individual income, average total population, and population of humans over 40 years old. Human‐made infrastructure consisted of variables describing the percentage of buildings, house density, percentage of roads, and percentage of other paved surfaces (such as sidewalks) in a grid cell. Natural habitat variables were percentage of meadows, percentage of city parks, percentage of grass and shrubs, percentage of forest, percentage of bare earth, number of trees, elevation, and percentage of tree canopy. Briefly, variation partitioning is calculated as the ratio of the sums of squared deviations of our explanatory variable to the total sums of squared deviations from the model (Legendre, 2007).

## 3 RESULTS

**FIGURE 3:**
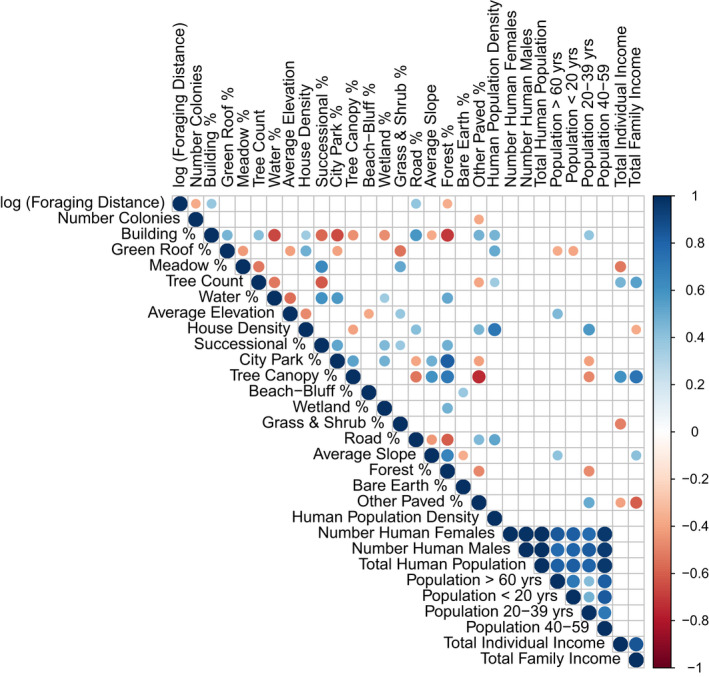
Correlation structure of environmental, demographic, and population parameters. Only statistically significant correlations are depicted (after multiple testing correction), and the size and color of the dots represent the value of the Spearman correlation coefficient. Table 2 contains an appendix of terms

The mean foraging distance per 4 km^2^ grid cell was 976 ± 268 (SE) meters, while the mean effective number of colonies per grid cell was 2.66 ± 0.28 (SE). Bumblebee colony density and foraging distance were negatively correlated (*r* = −.37, *p* = .04, Figure 3) at the grid cell level. The pairwise Spearman correlation analysis between all physical and demographic features of Toronto suggested several variables that influence *B*. *impatiens’* colony density and foraging distance (Figure 3). Using this simple analysis, we found that the relative area of “other paved surfaces” was negatively associated with the bumblebee colony density (*r* = −.38, *p* = .03, Figure 3). The mean foraging distance was higher in cells with more buildings (*r* = .39, *p* = .03), and roads (*r* = .39, *p* = .02), and lower in cells with more watershed forests (*r* = −.37, *p* = .04 Figure 3). Bumblebee colony density and foraging distance were not correlated with any of Toronto's human population demographic variables (Figure 3).

**FIGURE 4:**
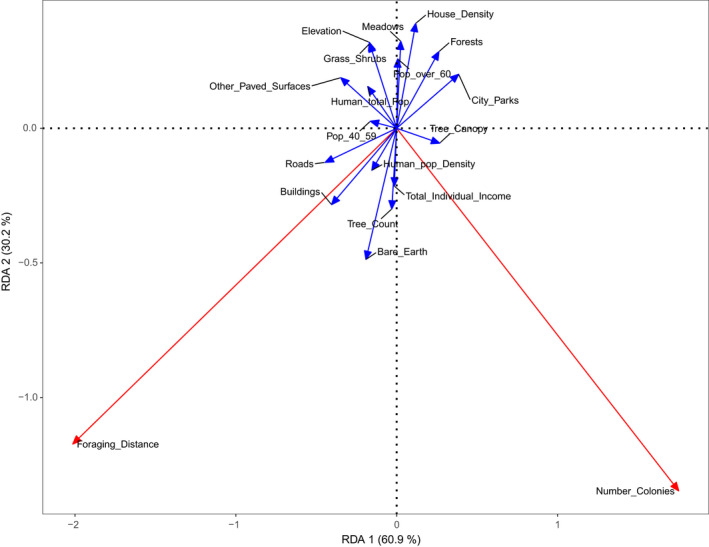
A redundancy analysis (RDA) for bumblebee foraging distance and effective number of colonies explained 55.2% of variation with an adjusted *r* ^2^ of 30.8% based on explanatory variables accounting for natural habitat, human‐made infrastructure, and human population and demographic factors. RDA axis 1 explains 33.6% of the variation and RDA axis 2 explains 16.7% of the variation. The arrows in blue represent the explanatory variables, and the variables in red are the response variables. Notes: Grass/Shrub % has the same direction and weight as Elevation. Average foraging distance was log‐transformed before analysis (see methods). Table 2 contains an appendix of terms

The redundancy analysis explaining the association between foraging distance and the number of unique bumblebee colonies, based on a set of environmental and human demographic variables, explained 55.2% of the variation in bumblebee nest density and bumblebee foraging distance (permutation test, *p* = .009, Figure 3). Proportionally, RDA axis 1 accounted for 69.1% of the variation in the model (33.6% of the total variation), while RDA axis 2 explained 30.9% of the variation (16.7% of the total variation). Following established practices (Borcard et al., 2011; Legendre & Legendre, 2012), we compared the vector loadings of the explanatory variables in the RDA analysis to those of the response variables to gain insights on the landscape and demographic factors that influence bumblebee habitat quality in Toronto (Figure 4). The RDA analysis indicated that bumblebee foraging distance is higher in areas with a higher percentage of buildings, roads, population density, bare earth, total individual income, and tree count. Conversely, bumblebee foraging distance is lower in areas with a higher percentage of watershed forests, city parks, and residential housing. The number of unique bumblebee colonies was higher in areas with a higher percentage tree canopy, total individual income, and tree count and lower in areas where there are more paved surfaces and higher human population. Variation partitioning on the RDA revealed that natural environments explained most of the variation (35.1%), followed by human demographic factors (26.1%), and human infrastructure (26.0%). 12.7% of the variation was shared among the three categories.

In addition to our RDA models, we also carried out a series of multiple linear regressions to explore the combination of environmental parameters associated with the two genetic parameters representing high‐quality bumblebee habitat. The effective number of colonies per grid cell was best predicted by a model (*r* ^2^ = 17.6%, Table 3) with the following 6 explanatory variables: relative area of aesthetic green space (grass/shrubs) (variance explained = 5.05%), relative area of roads (variance explained = 4.54%), “other” paved surfaces (variance explained = 4.36%), and relative area of watershed forests (variance explained = 3.38%) were all negatively associated with colNe, while city parks (variance explained = 7.09%) and relative area of watershed meadows (variance explained = 3.46%) all exhibited a positive relationship with colNe (Table 3). The log of mean foraging distance within a grid cell was best predicted by a model (*r* ^2^ = 35.0%) with four explanatory variables (Table 4): relative area of roads (variance explained = 15.5%), bare earth percentage (variance explained = 5.95%), and “other” paved surfaces (variance explained = 0.11%) were positively associated with foraging distance, while house density (variance explained = 18.8%) was negatively associated with foraging distance (Table 4).

**TABLE 3:** Model selection for best 10 multiple linear models predicting the effective number of colonies within a grid

Model	Adjusted r^2^ (%)	AIC
7.67 −0.11*grass_shrub_percentage −0.14*road_percentage −0.09*other_paved_percentage −0.17*forest_percentage +0.12*city_park_percent +0.15*meadow_percentage	17.6	203.5
6.06 + 2.57*road_percentage −0.0*tree_count +0.07*tree_canopy_percentage −0.08*grass_shrub_percentage −0.2*road_percentage −0.08*other_paved_percentage −0.27*forest_percentage +0.15*city_park_percent +0.11*meadow_percentage	17.1	206.1
5.69 + 2.86*road_percentage −0.0*tree_count +0.07*tree_canopy_percentage −0.05*grass_shrub_percentage −0.2*road_percentage −0.08*other_paved_percentage −0.25*forest_percentage +0.14*city_park_percent	17.0	205.4
8.66 −0.09*grass_shrub_percentage −0.14*road_percentage −0.09*other_paved_percentage −0.01*elevat −0.15*forest_percentage +0.09*city_park_percent +0.13*meadow_percentage	16.9	204.7
7.59 −0.11*grass_shrub_percentage +0.19*bare_earth_percentage −0.14*road_percentage −0.09*other_paved_percentage −0.16*forest_percentage +0.11*city_park_percent +0.15*meadow_percentage	16.6	204.9
6.95 + 1.24*road_percentage −0.1*grass_shrub_percentage −0.2*road_percentage −0.08*other_paved_percentage −0.16*forest_percentage +0.12*city_park_percent +0.15*meadow_percentage	16.6	204.9
7.95 + 2.17*road_percentage −0.0*tree_count −0.1*grass_shrub_percentage −0.22*road_percentage −0.11*other_paved_percentage −0.17*forest_percentage +0.11*city_park_percent +0.12*meadow_percentage	16.3	205.9
6.54 −0.0*tree_count +0.08*tree_canopy_percentage −0.11*grass_shrub_percentage +0.09*building_percentage −0.12*road_percentage −0.12*other_paved_percentage −0.29*forest_percentage +0.16*city_park_percent +0.16*meadow_percentage	16.2	206.6
7.67 −0.12*grass_shrub_percentage −0.13*road_percentage −0.09*other_paved_percentage −0.17*forest_percentage +0.12*city_park_percent +0.17*meadow_percentage −0.46*wetland_percentage	16.2	205.1
7.59 + 2.46*road_percentage −0.0*tree_count −0.06*grass_shrub_percentage −0.22*road_percentage −0.11*other_paved_percentage −0.15*forest_percentage +0.1*city_park_percent	16.2	205.2

**TABLE 4:** Model selection for best 10 multiple linear models predicting the mean foraging distance of colonies within a grid

Model	Adjusted r^2^ (%)	AIC
3.43 + 2.16*roads_percentage +0.42*bare_earth_percentage +0.08*other_paved_percentage ‐ 0.0001*house_density	35.0	187.5
4.07 + 0.11*building_percentage +0.59*bare_earth_percentage ‐ 0.0001*house_density	32.6	188.4
3.65 + 1.97*road_percentage +0.4*bare_earth_percentage +0.09*other_paved_percentage ‐ 0.0001*house_density ‐ 0.06*meadow_percentage	34.8	188.6
3.75 + 0.55*bare_earth_percentage +0.08*building_percentage +0.08*road_percentage ‐ 0.0*house_density	33.1	188.9
2.86 + 2.49*roads_percentage +0.42*bare_earth_percentage +0.09*other_paved_percentage +0.02*forest_percentage ‐ 0.0001*house_density	34.1	189.0
3.12 + 2.52*roads_percentage +0.46*bare_earth_percentage +0.08*other_paved_percentage +0.07*forest_percentage ‐ 0.0001*house_density ‐ 0.06*city_park_percentage	35.2	189.1
3.27 + 2.4*road_percentage +0.09*other_paved_percentage ‐ 0.0001*house_density	31.3	189.3
3.96 + 0.55*bare_earth_percentage +0.1*building_percentage +0.04*other_paved_percentage ‐ 0.0001*house_density	32.5	189.3
3.23 + 1.88*roads_percentage +0.0*tree_count +0.44*bare_earth_percentage +0.09*other_paved_percentage ‐ 0.0001*house_density	33.7	189.3
3.49 + 0.62*bare_earth_percentage +0.09*building_percentage +0.1*road_percentage +0.09*forest_percentage ‐ 0.0*house_density ‐ 0.08*city_park_percentage	34.8	189.4

## 4 DISCUSSION

Our high‐resolution analyses of the landscape features that enhance pollinator habitats in urban environments in Toronto are consistent with previous studies on bumblebees and other generalist native bees. Specifically, previous studies linked impervious paved surfaces and availability of forage as key negative and positive drivers, respectively, of habitat quality for native bee communities (Bennett & Lovell, 2019; Birdshire et al., 2020; Carvell et al., 2011; Egerer et al., 2020; Fortel et al., 2014; Geslin et al., 2016; Goulson et al., 2010; Jha & Kremen, 2013a, 2013b,2013a, 2013b).

In our correlation analysis, the effective number of colonies (colNe) within a grid cell was negatively associated with the relative area of “other paved surfaces” (Figure 3). Both the RDA analysis (Figure 4) and the multiple linear regression analyses (Table 3) also showed the benefits of green space and the disadvantages of man‐made structures on colony density. These results are intuitive given that *B*. *impatiens* primarily nest in underground cavities that are often near trees and woody shrubs (Lanterman et al., 2019).

Like colony density, the average foraging distance was also impacted by the degree of urbanization within Toronto. Our correlation analysis showed that more buildings and roads were associated with greater foraging distances (Figure 3). The RDA (Figure 4) and multiple linear regression analyses (Table 4) supported and expanded on the simpler analysis. In our RDA model (Figure 4), bumblebee foraging distance increased with higher densities of buildings, roads, paved surfaces, bare earth, and human population size (i.e., greater urbanization). Grid cells with more forests, city parks, and residential housing were associated with lower foraging distance. Our best fitting multiple linear regression model (Table 4) recapitulated the negative influence of urbanization on foraging distance: Both the relative area of roads, other paved surfaces, and bare earth were associated with higher foraging distances. Our findings are consistent with previous research showing that human structures, such as roads and railroads, substantially restrict foraging by bumblebees workers who avoid flying across these man‐made structures (Bhattacharya et al., 2003). Surprisingly, the relative density of houses within grid cells was associated with shorter foraging distances. This likely reflects that the type of urbanization matters. In other words, in the absence of city parks or watershed forests, urban areas with a higher density of single or multiple family houses likely provide better foraging opportunities for bumblebees relative to urban areas with a high density of multilevel buildings. Houses in Toronto typically have a front yard and a back yard, which can provide some foraging opportunities for bumblebees throughout their active flight season (e.g., flowering trees, small‐scale gardens, and weeds).

There has been conflicting evidence on the role of the “luxury effect” (Leong et al., 2018) in pollinator conservation. One study in the city of Chicago (USA) showed that lower income neighborhoods faired similarly in terms of bee abundance and diversity relative to higher income neighborhoods (Lowenstein et al., 2014). However, a UK study showed that higher household income was positively associated with pollinator abundance (Baldock et al., 2019). Our bivariate analysis indicated that income was not associated with either foraging distance or colony density. Our study in Toronto appears to be more in line with Chicago (both in North America), where income did not appear to influence bumblebee habitat quality. We think this bodes well for ensuring that Toronto's diverse community benefits equally from the pollination services that bumblebees, and possibly other wild pollinators, provide. Lack of a “luxury effect” in Toronto also implies that the opportunities for observing bumblebees and engaging in bumblebee conservation is—in theory—not restricted to a subset of Toronto's population.

While it may be impossible to fully generalize our analysis of a single species to the entire pollinator community, we have several lines of evidence that suggest that landscape features that improve habitat quality for *B*. *impatiens* would also improve habitat for *many* other native bees living in Toronto as well. Like the majority of bees found in cities (MacIvor et al., 2014; Matteson et al., 2008), *B*. *impatiens* is a diet generalist that is known to forage on a large number of plant species (Colla & Dumesh, 2010; Gervais et al., 2020; Vaudo et al., 2014), including many that attract other native pollinators. For example, in Southern Ontario, *B*. *impatiens* visits species from 70 plant genera, including maple (*Acer* sp.), black‐eyed Susan (*Rudbeckia hirta*), Canadian goldenrod (*Solidago canadensis*), New England aster (*Symphyotrichum novae*‐*angliae*), purple coneflower (*Echinacea purpurea*), Willow (*Salix* sp.), and trees and shrubs in the genus *Prunus* to name a few; all highly attractive to other native pollinators. We thus expect that areas in Toronto that promote shorter foraging distances for *B*. *impatiens* would also provide foraging opportunities for other native bees. Additionally, our analysis on *B*. *impatiens* has implicated similar features previously known to influence habitat quality for native bees. Impervious structures (e.g., roads, buildings, and other paved surfaces) had a clear negative impact on both *B*. *impatiens*’ colony density and foraging distance in our analysis; similar detrimental effects of impervious surfaces have been documented for other bumblebee species (Jha & Kremen, 2013a, 2013b,2013a, 2013b) and native bee communities (Bennett & Lovell, 2019; Birdshire et al., 2020; Egerer et al., 2020; Fortel et al., 2014; Geslin et al., 2016). Similarly, functional green space in Toronto was associated with shorter foraging distances in our RDA analysis, which is consistent with previous research on the importance of forage for many other native bumblebees (Carvell et al., 2011, 2012, 2017; Goulson et al., 2010; Redhead et al., 2016). We thus think it is reasonable to assume that our analysis on *B*. *impatiens* can help us identify high‐quality habitat for other native pollinators given that: (1) landscape features associated with high‐quality habitat for *B*. *impatiens* in Toronto have also been associated with high‐quality habitat for native bee communities elsewhere; (2) *B*. *impatiens* workers visit a diverse assemblage of plants that attract and provide resources to wide assemblages of native bees.

Overall, our study shows the importance of functional green space in providing high‐quality habitat for bumblebees in an urban environment such as the city of Toronto. Both colony density and foraging distance were influenced by the degree of urbanization in Toronto. Impervious paved surfaces (e.g., roads, buildings, and other paved surfaces) were associated with differences in colony density and foraging distances. While green space is important for bumblebees, our study indicated that natural/functional green spaces, such as city parks and forests, were often beneficial relative to aesthetic green spaces, such as lawns. Our analysis suggests two simple strategies for improving bumblebee habitat within cities. First, conversion of paved surfaces to functional green space such as parks and meadows is likely to have a significant influence on the quality of pollinator habitats in Toronto. Second, our RDA (Figure 4) suggests that converting aesthetic green space (i.e., lawns, which is orthogonal to the foraging distance vector and is opposite to the colony density vector) into more functional natural green space (e.g., flowering meadows, which tend to be associated with lower foraging distances) can improve the foraging opportunities of bumblebee colonies in Toronto.

## CONFLICT OF INTEREST

None declared.

## AUTHOR CONTRIBUTIONS

**Ida M. Conflitti:** Conceptualization (supporting); Formal analysis (equal); Methodology (lead); Writing – original draft (equal); Writing – review & editing (equal). **Mohammad Arshad Imrit:** Formal analysis (equal); Methodology (supporting); Writing – original draft (equal); Writing – review & editing (equal). **Bandele Morrison:** Formal analysis (supporting); Methodology (supporting); Writing – review & editing (equal). **Sapna Sharma:** Formal analysis (supporting); Methodology (supporting); Supervision (equal); Writing – original draft (equal); Writing – review & editing (equal). **Sheila R. Colla:** Conceptualization (equal); Supervision (equal); Writing – original draft (equal); Writing – review & editing (equal). **Amro Zayed:** Conceptualization (equal); Funding acquisition (lead); Supervision (equal); Writing – original draft (equal); Writing – review & editing (equal).

## Supporting information

Data S1

## Data Availability

All raw and processed data can be found as Data S1. The code for carrying out the statistical landscape analyses can be downloaded on Github (https://github.com/mimrit/Toronto_Bumble_Bees).
